# RESOURCE CENTERS FOR MINORITY AGING RESEARCH (RCMAR) SUCCESS AND OPPORTUNITY

**DOI:** 10.1093/geroni/igae098.0680

**Published:** 2024-12-31

**Authors:** Carmen Sceppa

**Affiliations:** Chair

## Abstract

The Resource Centers for Minority Aging Research (RCMAR) have been continuously funded by the National Institute on Aging (NIA) since 1997. The RCMAR program offers intellectual leadership, mentorship, training, career development opportunities, and pilot study funding for future leaders in aging research in the behavioral and social sciences who are at an early stage in their careers. The Gerontological Society of America (GSA) – the nation’s largest interdisciplinary organization devoted to the field of aging – was awarded a $3.9M cooperative agreement from the NIA (2023-2028) to run the RCMAR VI National Coordinating Center (NCC) with a multiple PI leadership team including Patricia M. D’Antonio, GSA vice president of policy and professional affairs, executive director of the National Center to Reframe Aging; Tamara A. Baker, professor, Department of Psychiatry, School of Medicine, University of North Carolina at Chapel Hill; Lisa L. Barnes, the Alla V. and Solomon Jesmer Professor of Gerontology and Geriatric Medicine and cognitive neuropsychologist, Rush Alzheimer’s Disease Center; Carmen Sceppa, dean, Bouvé College of Health Sciences, professor of health sciences, Northeastern University; and James C. Appleby, GSA chief executive officer. There are 18 RCMAR Centers in the U.S., 10 focused on Alzheimer’s disease (AD) and AD-related dementias (ADRD) and 8 aging-focused. The RCMAR mission is to bolster mentorship and career development of scientists from diverse backgrounds and foster rigorous behavioral and social science research to advance aging-relevant scientific discoveries, eliminate health disparities, improve the health and well-being of older adults, per NIA’s Health Disparities Research Framework.

